# *Culex quinquefasciatus* larval microbiomes vary with instar and exposure to common wastewater contaminants

**DOI:** 10.1038/srep21969

**Published:** 2016-02-25

**Authors:** Marcus J. Pennington, Sean M. Prager, William E. Walton, John T. Trumble

**Affiliations:** 1 Department of Entomology, University of California, Riverside, USA; 2 Graduate Program in Environmental Toxicology, University of California, Riverside, USA

## Abstract

Like many insects, mosquitoes, rely on endosymbionts to grow and develop. These can be acquired from the environment. We used next generation 454 pyrosequencing to discern the whole-body microbiome of the mosquito species *Culex quinquefasciatus* in various larval stadia and following exposure to common pharmaceutical and personal care products (PPCPs) found in wastewater. PPCP treatments included environmentally-relevant concentrations; 1) a combination of common antibiotics, 2) a combination of mammalian hormones, 3) a mixture of the antibiotic and hormone treatments plus acetaminophen and caffeine and, 4) an untreated control. Within control groups, the predominant families of bacterial symbionts change with each larval instar despite consistent diets and rearing conditions. This trend was also seen in hormone treatments but not in the antibiotic or the mixture treatments. Richness and evenness were reduced in both antibiotic and mixture treatments, suggesting that antibiotics remove certain bacteria or inhibit them from increasing to proportions seen in the control treatment. Interestingly, the mixture treatments had greater richness and evenness compared to antibiotic alone treatments, possibly due to the other contaminants facilitating growth of different bacteria. These findings illuminate the complexity of the microbiome of *C. quinquefasciatus* and may have implications for more effective control strategies.

Endosymbionts, bacterial species known to grow, develop, and vertically transmit in an organisms’ cells, usually for mutualistic symbiosis, are essential to the growth, development, and fecundity of many insect species^1^,^2^,^3^,^4^,^5^. Many aphid species, such as *Acyrthosiphon pisum, Megoura viciae*, and *Myzus persicae*, rely heavily on endosymbionts to survive on the unbalanced diet of phloem sap. Their bacteriocyte endosymbiont, *Buchnera spp*, provides essential amino acids^6^. Without *Buchnera*, aphids demonstrate reduced growth rates and development and produce few or no offspring^6^,^7^. However, some endosymbionts in the genus *Wolbachia* are known to manipulate insects for their own benefit and can also lead to increased vector competency for transmission of human diseases such as West Nile Virus^8^,^9^. *Wolbachia* species are common vertically transmitted endosymbionts in many mosquito species and typically infect reproductive tissue^10^. There are many species of *Wolbachia* that influence a variety of insect behaviours and life-history traits. Some endosymbionts can also act defensively by killing parasitoid eggs after they are laid in the host^8^,^10^,^11^. Because endosymbionts play major roles in insect development, they have been proposed for use in insect control^12^.

Due to the importance of endosymbionts for development in many insect species, some species vertically transfer endosymbionts (from parent to offspring). For example, Estes *et al.*^13^ describes the microbiome of dung beetles’ (*Onthophagus taurus*) brood balls, which are used to nourish their offspring until they are adults. When the microbiota in the beetle offspring and their female parent were compared in sterilized dung and soil, they had proportionally identical 16S rRNA sequences from their microbiome. Interestingly, over their life stages, the proportions of the families of bacteria in the beetles’ microbiome changed. The predominant family of the first three instars varied by individual. However, from the pupal stage onwards, *Enterobacteriaceae* was the most common family in the dung beetle. Bees acquire important bacteria through social interaction and also from the environment^14^. Without some of these bacteria, it is thought that honey bees could become more susceptible to outside diseases and increase incidents of colony collapse^15^. More studies are needed to fully understand the importance of microbiota in mosquitoes as they have been linked to increased transmission of pathogens from mosquitoes to humans^9^,^16^.

Mosquitoes are common disease vectors, which spend their juvenile stages in aquatic environments^17^. Bacteria from the water, both symbiotic and free-living, have been shown to influence the microbiome of mosquitoes, suggesting that some of their possible endosymbionts are collected from the environment^18^. Consequently, if the environment is altered, and some of these necessary bacteria are reduced/eliminated, there could be detrimental effects on the development of mosquito larvae. Such a removal effect may occur as the result of antibiotic runoff or other pollution, and/or environmental changes. For example, Rosi-Marshall *et al.*^19^ showed that common pharmaceuticals in streams will alter the respiration and diversity of biofilms. Pennington *et al.*^20^ reported differences in whole-body microbiomes and increased developmental times for *Culex quinquefasiatus* (Say) treated with various pharmaceuticals and personal care products (PPCPs).

Chemicals intended for human use often occur in aquatic environments and/or enter water supplies through water treatment plant overflow or from use of reclaimed water in water scarce areas^21^,^22^,^23^,^24^,^25^,^26^. These chemicals can then affect bacterial communities in the water and the associated aquatic insect community. Presence of these contaminants can alter effectiveness of *Bacillus thuringiensis* subsp. *israelensis* (Bti) a pesticide commonly applied for mosquito control^20^. However, little is known about how such contaminants will influence the microbiome of such insects. Similarly, there is a lack of data determining if mosquito bacterial communities vary during the course of larval development; all available studies we are aware of examine only late instar larvae or mixed lower instars and species^5^,^27^, and information regarding mosquitoes’ endosymbionts and their function is very limited or non-existent outside of *Wolbachia*. Therefore, we describe the differences in the bacterial communities of the mosquito *C. quinquefasciatus* in multiple distinct larval stages, as well as when these mosquitoes are reared in environments contaminated with ecologically relevant concentrations of PPCPs that commonly occur in combinations in order to provide a baseline for more in-depth studies.

## Results

The mosquitoes’ microbiomes were significantly affected by both PPCPs (PERMANOVA: F = 3.78; df = 3, 32; p < 0.001) and instar (PERMANOVA: F = 8.64; df = 2, 33; p < 0.001). Additionally, there was a significant interaction of PPCP treatment and instars (PERMANOVA: F = 7.63; df = 6, 29; p < 0.001). The microbiome of the control mosquitoes changed significantly between instars (PERMANOVA: F = 10.39; df = 2, 8; p < 0.01). In pairwise comparisons of only larvae from the control treatments, second instar larvae were significantly different from third instar (p < 0.01) but were not different than fourth instars (p = 0.1) while, third and fourth instars were not significantly different (p = 0.1014) from each other. Families with at least 1% proportionality of the control treated mosquitoes’ microbiome in at least one instar were also examined via pairwise comparisons. The results are included in Table 1.

When PPCP treatments were examined for differences among instars, second (PERMANOVA: F = 4.05; df = 3, 8; p < 0.001), third (PERMANOVA: F = 10.25; df = 3, 8; p < 0.001), and fourth (PERMANOVA: F = 9.63; df = 3, 8; p < 0.001), were significantly affected by PPCP treatments. When subjected to pairwise comparisons, antibiotic and hormone treated mosquitoes’ microbiomes were significantly different between second and fourth instar only (p < 0.01), and there was no significant differences in any instars in the mixture treated mosquitoes (p = 0.3736).

**Figure 1:**
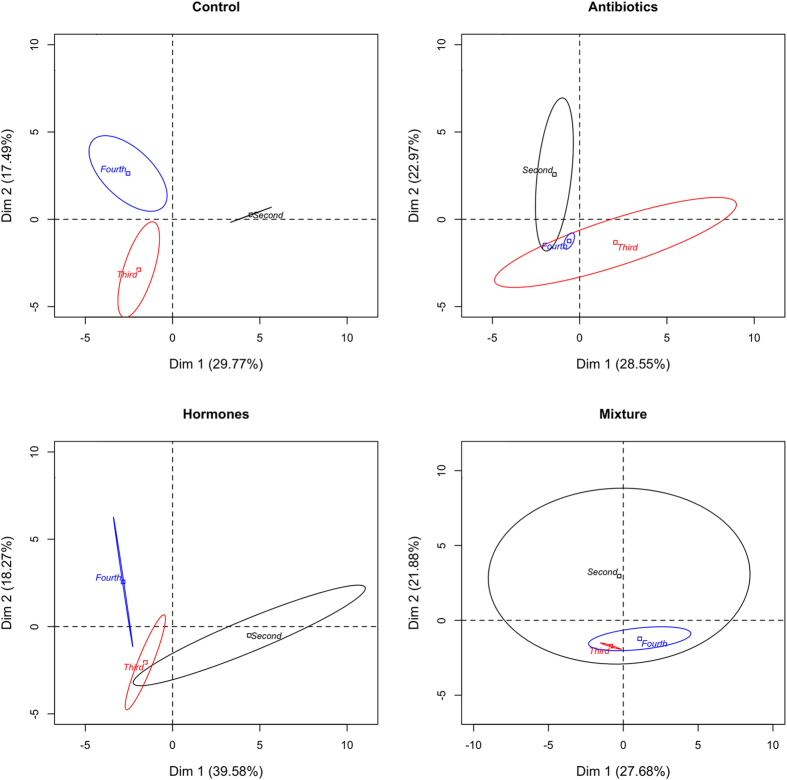
Principle component analysis of control and PPCP treatments by instar. Ellipses incorporate all instar replicates (n = 3) with the centre of the ellipses displaying the mean analysis.

**Figure 2:**
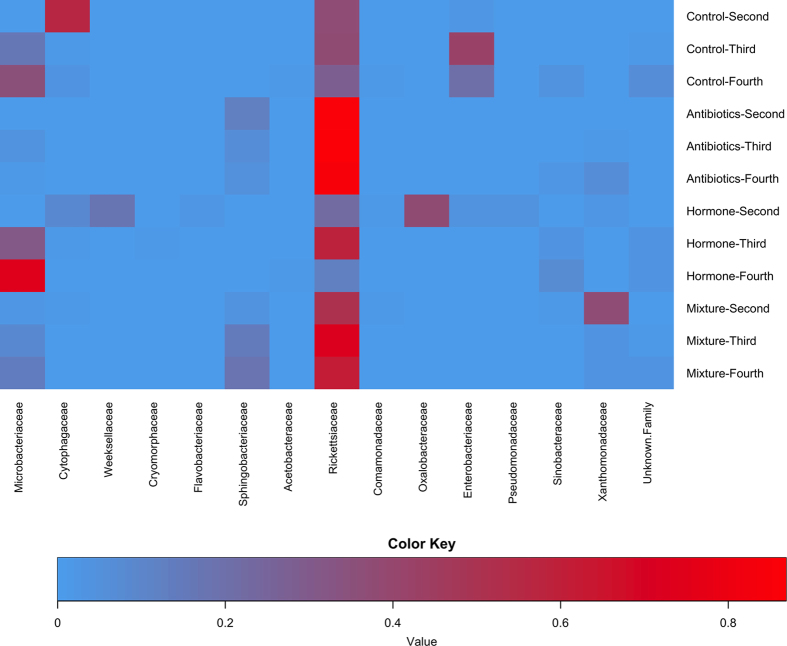
The top 15 families with relative proportions ≥1% by family and samples by PPCP treatment- instar on the X and Y-axes respectively. More predominant families appear darker on the map.

PCA (Fig. 1) demonstrates that various instars separate from each other in the four treatment groups with third and fourth instar loading similarly on the first dimension and all three instars loading distinctly in the second dimension. Bacterial families *Oxalobacteraceae* and *Aeromonadaceae* closely follow the separation pattern in the first dimension and *Cryomorphaceae* follows in the second (Table 2). In the antibiotic treatments second instar loaded separately from third and fourth on the second dimension (Fig. 1). The bacterial family *Propionibacteriaceae* also follows this trend and was the only family with at least 85% correlation in either of the first two dimensions (Table 2). For hormone treated mosquitoes, second instars loaded separately from fourth instars on the first dimension (Fig. 1). The bacterial families, which follow this trend, are *Enterobacteriaceae* and *Pseudomonadaceae*. As shown in Fig. 2 and Table 2, control treatments’ bacterial families changed with instar; starting with *Cytophagaceae* (56.25%) in second instars, changing to *Enterobacteriaceae* (41.81%) in third instar and finally the control fourth instars’ most predominant family was *Microbacteriaceae* (34.89%). There were a total of eight bacterial families with proportionalities greater then 1% in at least one instar of the control treatment. In fourth instars there is a resurgence of bacterial families from second instars, which were overshadowed by the third instar bacterial families (Table 1). Interestingly, the family *Rickettsiaceae* was the second most predominant family in all control instars. Operational taxonomic units (OTUs) assigned to the family *Rickettsiaceae* were found in most treatments and instars, although they were reduced in many hormone treated samples relative to the controls and increased in the antibiotic treatments. Notably, antibiotic and mixture treatment groups’ most predominant family was *Rickettsiaceae* over all instars. The second most predominant family in antibiotic and mixture treatment groups was *Sphingobacteriaceae* in all instars. Hormone treatments changed bacterial communities between instars but not as drastically as the control group. Second instars exposed to hormones predominately contained *Oxalobacteraceae*, which changed to *Microbacteriaceae* and *Rickettsiaceae* in the third instar. The predominant family of fourth instar hormones was *Microbacteriaceae*, although some proportion of *Rickettsiaceae* was still present.

**Figure 3:**
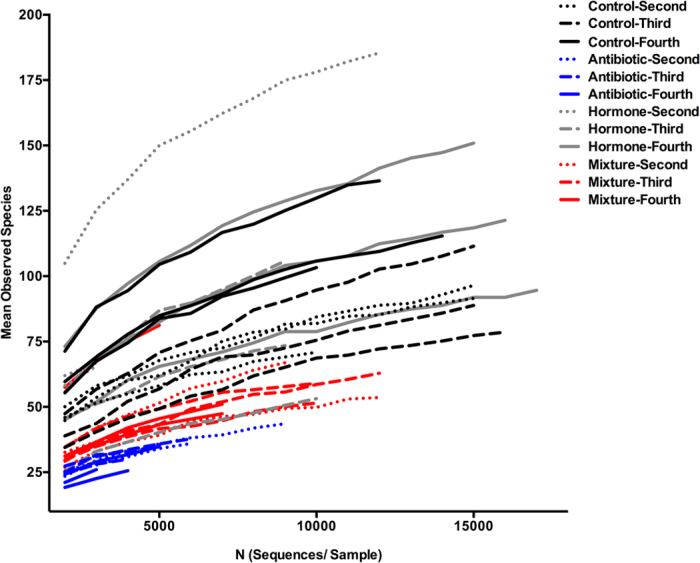
Mean observed species (n = 10 resampling events at a given subsampling interval) by the number of sequences per sample. Antibiotic, control, hormone and mixture treatments are shown by blue, black, grey, and red colours respectively. Dotted, dashed-dotted, and dashed line types represent second, third, and fourth instars respectively.

**Figure 4:**
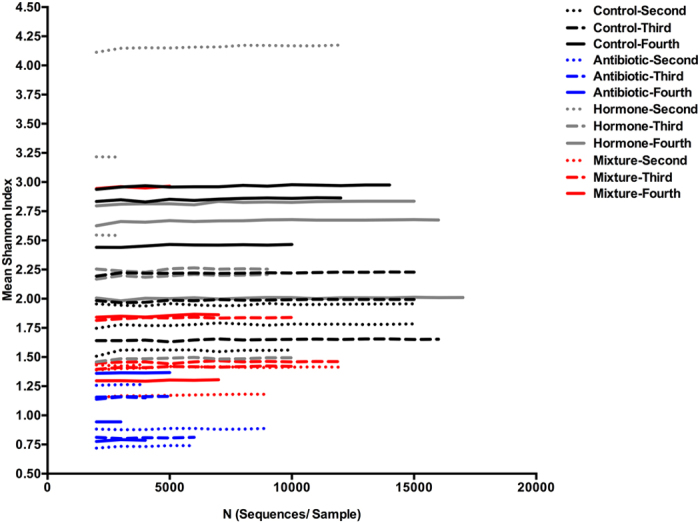
Mean Shannon’s index (n = 10 resampling events at a given subsampling interval) by the number of species sequences per sample. Antibiotic, control, hormone and mixture treatments are shown by blue, black, grey, and red colours respectively. Dotted, dashed-dotted, and dashed line types represent second, third, and fourth instars respectively.

In the alpha diversity analysis, richness (number of different species in one location) was examined as mean observed species (Fig. 3) and evenness (distribution of species’ proportionalities in one location) was measured by mean Shannon’s index (Fig. 4). For mean observed species at a sequencing depth of 3000 sequences/sample there was a significant difference between treatments (Χ^2^: 870.40; df: 3; p < 0.001), instars (Χ^2^: 80.02; df: 2; p < 0.001) and a significant interaction of treatment and instar (Χ^2^: 136.76; df: 6; p < 0.001). Mosquitoes treated with antibiotics had lower richness and fewer total sequences per sample than all other treatments with the richness decreasing as larvae age. This is evident in Fig. 2, as there are proportionally fewer bacterial families outside of *Rickettsiaceae* and *Sphingobacteriaceae* than in other treatments. In contrast, mosquitoes reared in the mixture of hormones, antibiotics and the common contaminants, acetaminophen and caffeine; demonstrate a relatively constant richness over time (Fig. 3). The control groups and hormone treatments fluctuate more than the mixture and antibiotic treatment groups but demonstrate consistently higher richness (Fig. 4). The mean Shannon’s diversity index suggests that antibiotics alone substantially reduced diversity. The mixture treatments also display reduced diversity, however, they are more diverse than their antibiotic treatment counterparts. The control groups display a greater diversity than both the antibiotic alone and mixture treatments when compared by increasing instar, where as the hormone treatment group, had no discernable pattern. The mixture also displays no discernable pattern compared by instar, which is likely due to the effects of the hormones added to antibiotics. Finally, it is notable that in some treatments, the mean species number (Fig. 3) failed to reach an asymptote. This may indicate that an inadequate sample size for detecting extremely rare species, or that the reduced diversity of some samples allowed for the entire community to be described.

## Discussion

Here we have demonstrated that the microbiome of larval *Culex* mosquitoes changes throughout development, and variation between instars is affected when exposed to various PPCPs. It has previously been demonstrated that mosquitoes rely on their microbiomes to aid in development and that removing certain symbionts can significantly slow larval development^5^,^28^,^29^. Pennington *et al.*^20^ demonstrated that PPCPs at environmentally relevant concentrations, which are significantly lower than those used in most laboratory studies, can alter the microbiome of mosquitoes and slow their development.

In the field, Duguma *et al.*^27^ showed pooled *Culex* species’ microbiomes will change from early (first and second) to late (third and fourth) instars. Coon *et al.*^5^ and Wang *et al.*^29^ showed that the microbiome of mosquitoes will change as the insects advance from fourth instar larvae, to pupae, to the adult stage, and after adults fed on a blood meal. We have shown that the microbiome of early (second and third) instars’ will change from one instar to the next even without exposure to PPCPs (control group). In the second, third, and fourth instars, predominant families change from *Cytophagaceae* to *Enterobacteriaceae* and finally to *Microbacteriaceae*. However similar to Pennington *et al.*^20^, third and fourth instar were not significantly different and our findings also correlate to what Duguma *et al.*^27^ found in their laboratory reared *Culex tarsalis* late instars. However, as their third and fourth instars were pooled, only the *Enterobacteriaceae* family predominates. These families were all removed in the antibiotic and mixture treatments. Fourth instar larvae in the control group match what was described in *Aedes aegypti* by Coon *et al.*^5^. Coon *et al.*^5^ also described the microbiome of two other mosquito species (*Anopheles gambiae* and *Georgecraigius atropalpus*) during the fourth instar. Their microbiomes had different proportions of familial microbiota between each other and both were different from the findings in our *C. quinquefasciatus* fourth instars. To our knowledge ours is the first study to look at the microbiome changes of individual early instars in mosquitoes. This suggests the possibility of a new strategy for mosquito control targeting the critical microorganisms essential for development at specific stages. Specifically, additional research targeting key symbionts found in earlier instars would determine if the younger larvae can be controlled more effectively, as has been seen with pesticides such as Bti^30^.

A number of mosquitoes are common carriers of the bacterial genus *Wolbachia,* which usually acts as a reproductive parasite in the ovaries of the females, and is suspected to be in at least 20% of all insect species^31^. As in Pennington *et al.*^20^, *Rickettsiaceae*, the family containing *Wolbachia pipientis,* continuously holds the majority count of the antibiotic and mixture treatments’ microbiome. When the OTUs mapped to the family *Rickettsiaceae* was examined at the level of genus the predominant and sole genus detected was *Wolbachia*. *Rickettsiaceae* is vertically transmitted from mother to offspring^32^; however, for many of the other bacterial families present, it is difficult to discern the source or how they are incorporated into the insects’ microbiome. This is made further complicated since these traits may vary by species or genus and mapping OTUs to finer taxonomic levels was generally not possible. Similarly, it may be possible to determine some origins via comparison with the water in rearing pans over time, although there was no DNA found in water at the start of the experiments. However, our focus was not on the origin of bacterial species in these mosquitoes, and we do not have these data. Analyses or the microbial community in such pans would be an interesting follow-up study. Interestingly, *Enterobacteriaceae*, which includes the genus *Buchnera* and other common endosymbionts, is the predominant family of the third instars in the control treatment. For example, the gut symbiont of the plataspid stinkbug (*Megacopta punctatiss*) is phylogenetically similar to *Buchnera* species^33^. In potato psyllids (*Bactericera cockerelli*) various genera of the family *Enterobacteriaceae* have been reported in the life stages and faeces accounting for at least 21% of the microbiome^34^,^35^. *Enterobacteriaceae* is one of, if not the most important family of endosymbionts in the pea aphid (*Acyrthosiphon pisum*)^6^, and is commonly used in research regarding the effects of antibiotics on insect-symbiont interactions^7^. Similarly, Chouaia *et al.* (2012)^28^ described a slowing of larval development in *Anopheles* mosquitoes when they removed *Asaia* bacteria from the family *Acetobacteraceae*. However this family has one of the lowest proportionalities in all of the mosquitoes including control treated. This suggests it is not an endosymbiont of this *Culex* mosquito species. However, reports of the effects of other PPCPs on insect-symbiont interactions are rare.

It is interesting that the hormones found in wastewater from treatment plants (and in the hormone treatment groups) are all mammalian female sex hormones and would not be expected to affect bacteria. We would not expect an effect of these hormones on bacteria, as they have no endocrine system; nonetheless, substantial changes in the microbiome occurred in response to exposure to these hormones (Fig. 1). Similarly, caffeine and an antihistamine, would not be expected to effect biofilms, but were shown to repress respiration in stream biofilms^19^. We think there may be some influence the hormones have on bacterial gene expression however that is not in the scope of this paper and thus, specific effects for each PPCP or combination of contaminants will need to be determined from more experimental data.

The increased bacterial diversity during mosquito ontogeny could result from either bacterial replication during development or by acquisition through ingestion. In the hormone and control treatment groups, we are unsure if bacteria are lost during development, or if the change in bacterial diversity was caused by differential growth among taxa. Interestingly, in pairwise comparisons of the hormone treated mosquitoes (Table 2), the majority of the significant differences were between second and fourth instar larvae. The hormone treated mosquitoes also had the most families that were correlated to the first principal component at a minimum of 85%. Combined this suggests that the mosquitoes exposed to hormones had the most diverse microbial communities and that this diversity increases over time.

Mammalian hormones change the microbiome of *C. quinquefasciatus* mosquitoes and it is possible they are responsible for the increased richness and diversity seen in the mixture treatments compared to antibiotics alone; however more studies will need to be conducted to confirm this conclusion. Regardless, our results indicate that reclaimed wastewater has the potential to impact mosquito ecology. Considerably more research will be required to discern how mixtures of PPCPs could affect bacterial microbiomes for important medical pests. If similar results are found for agriculturally important insects (including either pests and/or beneficial insects) exposed to these emerging contaminants, additional research documenting the effects of increasing use of reclaimed water and associated changes to the insect microbiome will become even more important. Similarly, because insects are a critical food source for higher trophic level organisms in terrestrial surface waters, releases of PPCPs in aquatic environments have the potential to modify the ecology of these ecosystems.

## Materials and Methods

### Insect Rearing

*Culex quinquefasciatus* mosquito egg rafts from colonies continuously reared at the University of California, Riverside, CA were maintained using the methods of Wirth *et al.*^36^. Egg rafts were held in shallow porcelain pans (30 × 20 × 5 cm) containing 3 L Geyser® Natural Alpine Spring Water (C G Roxane, Olancha, CA) or spring water and one of three PPCP treatments (described below). All pans contained food and any biologic/inorganic materials from the food and mosquito waste. It was beyond the scope of this project to simulate all of the possible small concentrations of various organic materials that could appear in wastewater from different treatment plants. Thus, the growing environments were made as reproducible as possible while still containing organics from the diet. Egg rafts and larvae were maintained in an incubator (model 818: Precision Scientific Inc., Buffalo, NY) at 28 °C, approximately 70% RH, and a light: dark cycle of 16:8. Mosquitoes were fed 2 mL of a mixture of 4 g brewers yeast and ground mouse chow (1:3 wt: wt) rehydrated in 50 mL water every 3 d. PPCP concentrations (Table 3) were used as in Pennington *et al.*^20^, and based on concentrations found by Kolpin *et al.*^21^ and Mutiyar and Mittal^37^. The treatment groups examined were: a control consisting of only water, an antibiotic treatment of lincomycin, oxytetracycline, and ciprofloxacin (Alfa Aesar, Ward Hill, MA; purity ≥98%), a hormone treatment of estrone, 19-norethindrone, 17β- estradiol, and 17α- ethynylestradiol (Sigma-Aldrich, St. Louis, MO; purity ≥98%), and a mixture of all PPCPs plus acetaminophen (MP Biomedicals, LLC, Santa Ana, CA; purity ≥90%) and caffeine (Fisher Scientific, Hanover Park, IL; laboratory grade purity). Despite Pennington *et al.*^20^ demonstrating that acetaminophen and caffeine do not alter the microbiome, these were included in the mixture treatment because they can and do co-occur in wastewater with the other contaminants and we did not want to assume there were no possible joint effects. The extended data set showing acetaminophen and caffeine has been presented as Supplementary Fig. S1. Hydrochloric acid (Fisher Scientific; 12.1 M) and sodium hydroxide pellets (Sigma-Aldrich, St. Louis, MO) were used to prepare 1 M stock solutions for adjusting final pH in rearing pans to 7 ± 0.5.

### Extraction of Bacterial DNA

In preparation for sequencing, three mosquitoes from each PPCP treatment were collected as second, third and fourth instars, as first instars were too small to collect without damage, and twice washed with 95% ethanol to remove any external microorganisms. Larvae were then transferred to individual sterile 2 mL microcentrifuge tubes with 95% ethanol and stored at −60 ± 3 °C in an ultra cold freezer (Forma Scientific, Inc. Marietta, OH) until DNA extraction. DNA was extracted using a Qiagen DNeasy^®^ Blood and Tissue Kit following the manufacturers protocols amended as in Pennington *et al.*^20^. In addition to mosquitoes samples of water and water plus diet were also extracted using identical protocols, with the additional step of centrifugation at 2900 rpm in an IEC HN-SII tabletop centrifuge for 1 h to create a pellet. Upon extraction, nucleic acid concentration was quantified using a Nanodrop ND- 2000c Spectrophotometer (Cole-Palmer, Vernon Hills, IL), to confirm enough genetic material for sequencing. This process revealed no DNA in water or water and diet samples when no mosquitoes were present and thus they were not subjected to further analysis because any bacteria would have originated from the mosquitoes, their egg-rafts, or from the air after the water had been altered by the mosquitoes various biological processes.

Roche 454 bacteria barcoded amplicon pyrosequencing was performed by a commercial sequencing facility (Molecular Research LP MR DNA, Shallowater, TX). The procedure used the forward primer 27Fmod (GRGTTTGATCMTGGCTCAG) and the reverse primer 519Rmodbio (GTNTTACNGCGGCKGCTG) in a single-step 30 cycle PCR using HotStarTaq Plus Master Mix (Qiagen, Valencia, CA). PCR was performed using the following cycle conditions: 94 °C for 3 min, followed by 28 cycles of 94 °C for 30 s; 53 °C for 40 s, 72 °C for 1 min; and a final elongation at 72 °C for 5 min. After PCR, all amplicons were mixed in equal concentrations and purified using Agencourt Ampure beads (Agencourt Bioscience Corporation, MA). Samples were then sequenced with Roche 454 FLX titanium instruments and reagents following the manufacturer’s guidelines.

### Sequence Analysis Pipeline

Raw bacterial DNA sequences were analysed using a MacQIIME (version: 1.8.0-20140103) based pipeline^38^. Raw .fasta and .qual files were combined to .fastaq file by the convert_fastaqual_fastaq.py command and uploaded to NIH/NLM/NCBI Sequence Read Archive (SRA) under SRP study accession number: SRP067136. Barcodes and primers were trimmed using the split_libraries.py script with default settings. After barcode trimming, data was denoised and re-inflated using the default denoise_wrapper.py script settings and the default setting in the inflate_denoiser_output.py script. Following denoising, no samples contained any ambiguous nucleotides. The maximum, minimum, and mean number of sequences across all samples was 22128, 3102, and 10099.11 respectively, with the average length of the reads being 403.84 base pairs. Operational taxonomic units (OTUs) were chosen by the default 97% identity threshold, which roughly correlates to species^39^, via the UCLUST method as implemented in the pick_otus.py script^40^. Representative OTUs were chosen using the pick_rep_set.py script and default settings. The Greengenes reference database clustered at 97% identity was used to assign taxonomy using the assign_taxonomy.py script^41^. OTUs were counted and summarized using the make_otu_table.py and summarize_taxa.py scripts respectively. OTUs were aligned using the align_seqs.py and filter_alignment.py scripts, and used to build a phylogenetic tree (make_phylogeny.py). There were 658 distinct OTUs at the species level with 58 distinct families; 15 OTUs failed to match any contained within the database and could not be assigned taxonomically. Fifteen families were chosen by their proportionality being greater than or equal to 1% in at least one sample for the heatmap. The cut-off was chosen at 1% as this was assumed to be the minimum to influence larval development at that stage. For alpha diversity, multiple rarefactions were performed using the multiple_rarefaction.py script with the lowest rarefaction depth of 2000, the highest rarefaction depth of 21000, a step size of 1000, and a replicate number of ten, which normalizes the data at each depth. Alpha diversity was calculated using the alpha_diversity.py script with the metrics observed species (species richness) and Shannon Indices (evenness) from the raw data^40^,^42^,^43^. Alpha diversity data was not averaged between replicate mosquitoes as they have been averaged by resampling-replicates and the complications and validity of this is still being considered^44^,^45^. Metrics were summarized using the collate_alpha.py script.

### Statistical Analyses

Statistical analyses were performed using R (the R Foundation for Statistical Computing, version 3.1.1). Following processing through the QIIME pipeline, “Permutational MANOVA” (PERMANOVA) in the Vegan package^46^ was used to compare the OTU data (Supplementary dataset 1). Independent variables were instar (n = 3), PPCP treatment (n = 4) and the interaction of the two, with three replicates (n = 3) of each instar in each PPCP treatment and control (n = 36). PERMANOVA is analogous to MANOVA but is suited to address the non-normality that is commonly associated with count data in ecological community and genetic data^47^,^48^. Microbial community data were further examined via principal component analysis (PCA) performed in the FactoMineR package^49^. Ellipses in the PCA encompass the three mosquito replicates in each instar for that treatment. PCA and PERMANOVA were conducted on each instar in the individual PPCP and control treatment groups. Following PCA, variables were examined for their contributions and correlation to each of the first two dimensions. Those variables (OTUs) that were ≥85% correlated were included in subsequent pairwise comparisons by instar in their respective treatment. Generalized linear hypotheses was used to perform pairwise comparisons in the multcomp package^50^. P values were adjusted using the p.adjust command. Alpha diversity data was analysed using a negative binomial generalized linear models at a sequence depth of 3000 sequences/sample to normalize data to the highest number where all sample mosquitoes were present. The alpha level for all tests was 0.05.

## Additional Information

**How to cite this article**: Pennington, M. J. *et al.* *Culex quinquefasciatus* larval microbiomes vary with instar and exposure to common wastewater contaminants. *Sci. Rep.* **6**, 21969; doi: 10.1038/srep21969 (2016).

## Supplementary Material

Supplementary Figure 1

Supplementary Dataset 1

## Figures and Tables

**Table 1:** Mean percentage of bacterial families by instar with at least 1% proportionality in the relative control mosquito instar.

Bacterial Phylum	Bacterial Family	Percentage Second Instar	Percentage Third Instar	Percentage Fourth Instar
*Bacteroidetes*	*Cytophagaceae*^a^,^b^,^c^	56.25	1.19	3.33
*Actinobacteria*	*Microbacteriaceae*^a^,^b^,^c^	0.00	15.84	34.89
*Proteobacteria*	*Rickettsiaceae*	36.72	37.49	28.15
	*Enterobacteriaceae*^a^,^b^,^c^	1.84	41.81	20.10
	*Sinobacteraceae*^a^,^b^,^c^	0.04	0.53	3.95
	*Acetobacteraceae*^b^	0.00	0.11	1.05
	*Comamonadaceae*^a^,^b^	0.86	0.08	1.02
Various	Unknown Family^a^,^b^,^c^	0.0598	1.300	6.336
	Sum Percentages	95.77	98.35	98.83
	Mean Total OTU	13821.00	15773.00	12741.67

^a^Denotes significant difference between second and third instar.

^b^Denotes significant difference between third and fourth instar.

^c^Denotes significant difference between second and fourth instar.

**Table 2:** All bacterial families with at least 85% familial correlation to one of the first two PCA dimensions.

Treatment	Phylum	Family	Dim	Second-Third	Second- Fourth	Third-Fourth
Control	*Proteobacteria*	Oxalobacteriaceae	1	*	*	
		Aeromonadaceae	1	*	*	
	*Bacteroidetes*	Cryomorphaceae	2	*		
Antibiotics	*Proteobacteria*	Propionibacteriaceae	2	*	*	
Hormones	*Bacteroidetes*	Weeksellaceae	1	*	*	
		Flavobacteriaceae	1	*	*	*
	*Firmicutes*	Bacillaceae	1		*	
		Paenibacillaceae	1	*	*	
		Leuconostocaceae	1		*	
	*Proteobacteria*	Sphingomonadaceae	1	*	*	*
		Oxalobacteraceae	1		*	
		Enterobacteriaceae	1	*		
		Pseudomonadaceae	1	*		
		Xanthomonadaceae	1	*	*	
Mix	*Proteobacteria*	Enterobacteriaceae	1	*	*	*
	*Bacteroidetes*	Cytophagaceae	1			

^*^Denotes a significant difference between the instars.

**Table 3:** PPCP treatment group components and rates.

Contaminant	Rate (μg/L)	Reference
Antibiotic		
Oxytetracycline	72.90	21
Lincomycin	0.730	21
Ciprofloxacin	31,000	37
Hormone		
17α-Ethynylestradiol	0.831	21
17β- Estradiol	0.200	21
19- Norethindrone	0.872	21
Estrone	0.112	21
Mixture		
Acetaminophen	10.00	21
Caffeine	6.000	21
Antibiotics	As above	
Hormones	As above	
