# Supplementary material for: Culex quinquefasciatus larval microbiomes vary with instar and exposure to common wastewater contaminants
Source: Sci Rep. 2016 Feb 25;6:21969. doi: 10.1038/srep21969 (PMC4766396; doi:10.1038/srep21969)
Supplement: Supplementary Figure 1 [file srep21969-s1.pdf]

Culex quinquefasciatus larval microbiomes vary with instar and exposure to common wastewater contaminants

Marcus J. Pennington*, [a, b] Sean M. Prager, [a] William E. Walton, [a] John T. Trumble, [a, b]

[a] Department of Entomology, University of California, Riverside, USA

[b] Graduate Program in Environmental Toxicology, University of California, Riverside, USA

Title: Expanded Heatmap

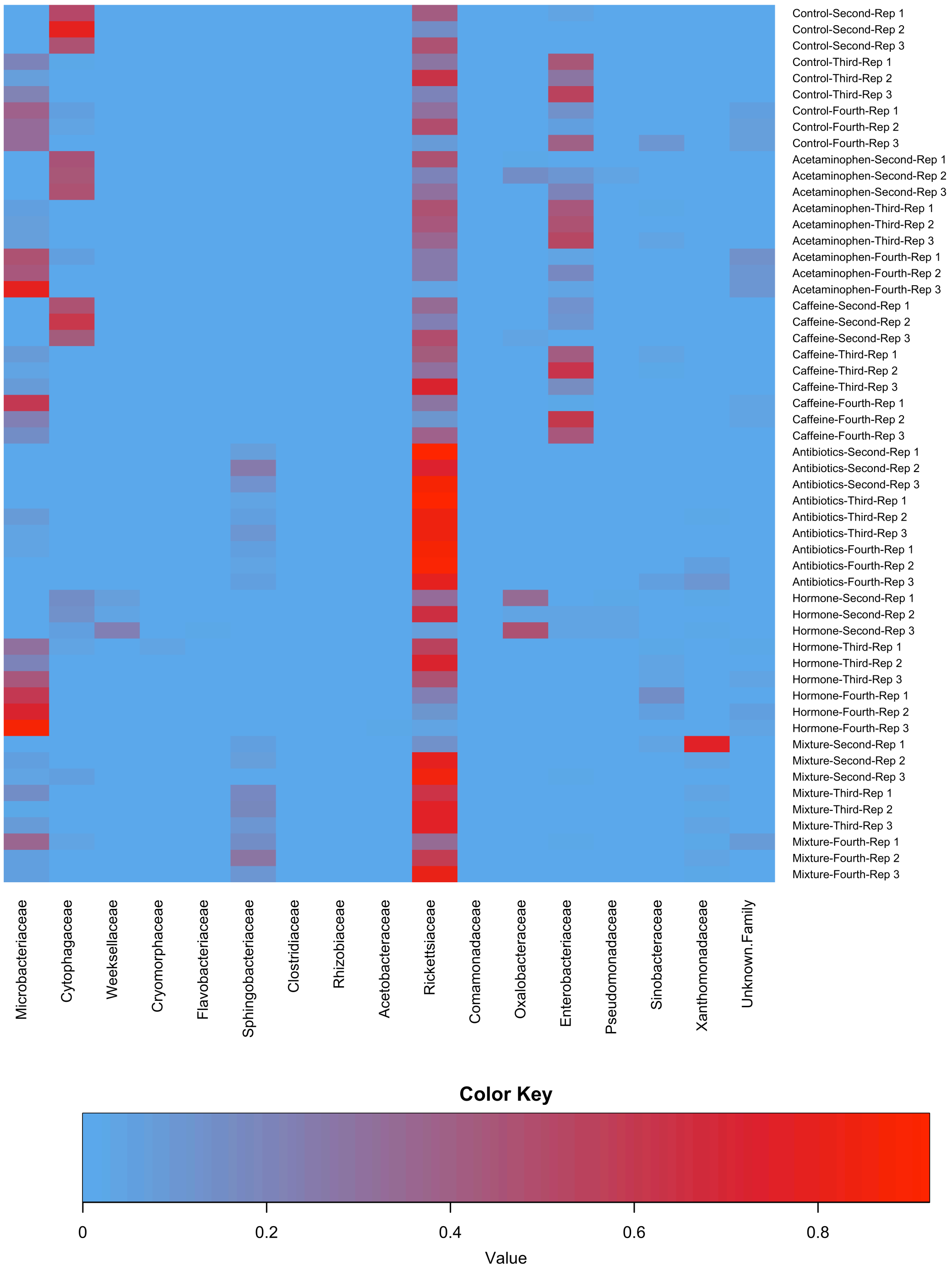

Supplementary Figure 1: The top 17 families with relative proportions ≥1% in at least one sample organized by PPCP treatment-instar-replicate on the X and Y-axes respectively. More predominant families appear redder.
